# The Antitumor Effect of Metformin Is Mediated by miR-26a in Breast Cancer

**DOI:** 10.3390/ijms17081298

**Published:** 2016-08-10

**Authors:** Paula Cabello, Begoña Pineda, Eduardo Tormo, Ana Lluch, Pilar Eroles

**Affiliations:** 1 Biomedical Research Institute INCLIVA, 46010 Valencia, Spain; paucanavarro@gmail.com (P.C.); bepime@hotmail.com (B.P.); eduardo.tormo@uv.es (E.T.); lluch_ana@gva.es (A.L.); 2 Oncology and Hematology Department, Hospital Clinico Universitario, 46010 Valencia, Spain

**Keywords:** miR-26a, metformin, breast cancer

## Abstract

Metformin, a drug approved for diabetes type II treatment, has been associated with a reduction in the incidence of breast cancer and metastasis and increased survival in diabetic breast cancer patients. High levels of miR-26a expression have been proposed as one of the possible mechanisms for this effect; likewise, this miRNA has also been associated with survival/apoptosis processes in breast cancer. Our aim was to evaluate if miR-26a and some of its targets could mediate the effect of metformin in breast cancer. The viability of MDA-MB-231, MDA-MB-468, and MCF-7 breast cancer cell lines was evaluated with an MTT assay after ectopic overexpression and/or downregulation of miR-26a. Similarly, the expression levels of the miR-26a targets *CASP3*, *CCNE2*, *ABL2*, *APAF1*, *XIAP*, *BCL-2*, *PTEN*, *p53*, *E2F3*, *CDC25A*, *BCL2L1*, *MCL-*1, *EZH2*, and *MTDH* were assessed by quantitative polymerase chain reaction (PCR). The effect of metformin treatment on breast cancer cell viability and miR-26a, *BCL-2*, *PTEN*, *MCL-1*, *EZH2*, and *MTDH* modulation were evaluated. Wound healing experiments were performed to analyze the effect of miR-26a and metformin treatment on cell migration. MiR-26a overexpression resulted in a reduction in cell viability that was partially recovered by inhibiting it. *E2F3*, *MCL-1*, *EZH2*, *MTDH*, and *PTEN* were downregulated by miR-26a and the PTEN (phosphatase and tensin homolog) protein was also reduced after miR-26a overexpression. Metformin treatment reduced breast cancer cell viability, increased miR-26a expression, and led to a reduction in *BCL-2*, *EZH2*, and *PTEN* expression. miR-26a inhibition partly prevents the metformin viability effect and the *PTEN* and *EZH2* expression reduction. Our results indicate that metformin effectively reduces breast cancer cell viability and suggests that the effects of the drug are mediated by an increase in miR-26a expression and a reduction of its targets, *PTEN* and *EHZ2* Thus, the use of metformin in breast cancer treatment constitutes a promising potential breast cancer therapy.

## 1. Introduction

Breast cancer is the most frequent cancer among women worldwide and the leading cause of death by cancer in women [1]. Breast cancer is a clinically, morphologically, and molecularly-heterogeneous disease [2,3]. In the year 2000, Perou et al. [4] classified it into five molecular subtypes according to its intrinsic genetic signature, however, immunohistochemical classification is still used in clinics. Treatment is based on the differential characteristics of breast cancer subtypes and is largely successful in human epidermal growth factor receptor 2 (HER2) and estrogen receptor (ER) positive (luminal) cancers (using anti-HER2^+^ and hormonal therapies, respectively). However, in triple negative breast cancer (TNBC; HER2, ER, and progesterone receptor negative), representing about 15%–20% of all breast cancer patients, there are no well-defined molecular targets. This subtype is related to an elevated recurrence rate, worse prognosis and a lower survival rate compared to other types of breast cancer [5,6] due, among other reasons, to the heterogeneity and aggressive nature of TNBC [6]. This peculiarity, and the fact that targeted treatments in HER2^+^ and ER^+^ breast cancers are not always beneficial, has led to the continued search for alternative therapies. However, extending the use of already approved drugs to new pathologies is a promising and rapid strategy for expanding the clinical therapeutic arsenal.

Metformin (1,1-dimetilbiguanide hydrochloride) is a hypoglycemic oral biguanide drug that is prescribed and commercialized worldwide to treat diabetes type II. Epidemiological studies have revealed that oral use of metformin has a protective effect against tumors, reducing their incidence and improving the prognosis of cancer patients [7,8]. This drug can inhibit cancer cell proliferation, although its molecular mechanisms of action are not yet completely understood [9].

MicroRNAs (miRNAs or miRs) are small non-coding endogenous RNA molecules (approximately 22 nucleotides long) encoded in the introns of protein-coding genes and in the introns and exons of non-protein coding genes, which regulate gene expression at the post-transcriptional level [10,11]. MicroRNAs have already proven to be reliable biomarkers for predicting therapeutic response in several cancers [12,13,14,15,16] or as therapeutic tools in other types of cancer, not only by inhibiting them with drugs [17] but also, for example, by introducing them into liposomal vehicles for systemic distribution in lung cancer or hepatocellular carcinoma model mice [18,19].

In a previous study from our group [20], we observed significant changes in miR-26a levels when treating breast cancer cell lines with doxorubicin. This miRNA has also been previously studied in other types of cancer including renal and lung cancer. In 2014, Yang et al. [21] demonstrated that metformin inhibits renal cancer cell growth by inducing overexpression of the oncogenic microRNA, miR-26a, which has *BCL-2* and *PTEN* among its targets. In this study we aimed to determine if miR-26a and/or some of its effector targets are implicated in the antitumor effect of metformin in TNBC and ER^+^ breast cancer, particularly in cell viability and/or migration.

## 2. Results

### 2.1. miR-26a Expression Modulates Cell Viability

**Figure 1:**
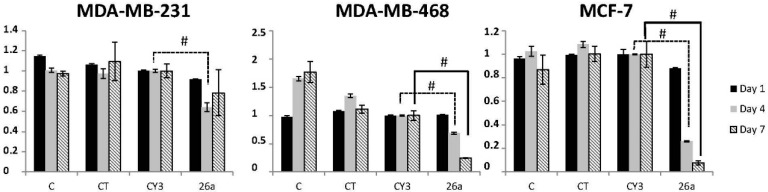
The effect of miR-26a on cell viability. MDA-MB-231, MDA-MB-468, and MCF-7 at days 1, 4, and 7 after transfection with miR-26a. C: non-treated control, CT: cells with transfection reagents, CY3: control with CY3 miRNA, 26a: miR-26a mimetic; 50 nM of pre-miRNA were transfected in all cases. Error bars represent the standard deviation of three experiments. Statistically significant differences comparing cells transfected with miR-26a to CY3 cells are shown at the respective time points. (Student *t* test: # *p* < 0.001).

We studied the effect of exogenous miR-26a on cell viability by transfecting three cell lines (MDA-MB-231, MDA-MB-468, and MCF-7) with a miR-26a mimetic. Compared to the miRNA control (Cy3), miR-26a overexpression decreased cell viability in all three cell lines at all of the time-points we assayed (1, 4, and 7 days), and the difference was statistically significant at four days (36% (*p* = 0.0004), 31.11% (*p* = 0.0009), and 73.89% (*p* = 4.7 × 10^−10^) decrease in viability for MDA-MB-231, MDA-MB-468, and MCF-7 cells, respectively) and 7 days (75.47% (*p* = 9.66 × 10^−5^), and 92.32% (*p* = 3.78 × 10^−6^) decrease in viability for MDA-MB-468 and MCF-7 respectively) (Figure 1).

**Figure 2:**
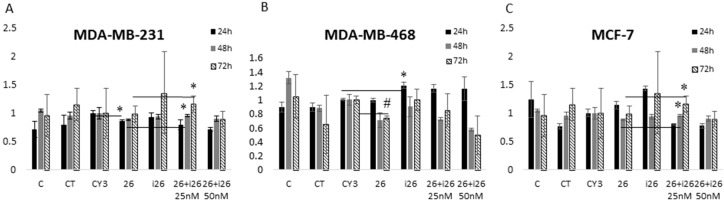
Determination of the miR-26a mimetic (26), the inhibitor (i26), and their combined effects on the viability of the (**A**) MDA-MB-231; (**B**) MDA-MB-468; and (**C**) MCF-7 cell lines at 24, 48, and 72 h after transfection; C: non-treated control, CT: cells with transfection reagents, CY3: control CY3 miRNA, 26: cells treated with 50 nM miR-26a mimetic; i26: cells treated with 50 nM miR-26a inhibitor, 26 + i26 is a combination of 25 + 25 nM or 50 + 50 nM. Error bars show the standard deviation of three experiments. (Student *t* test: * *p* < 0.05; # *p* < 0.001).

However, rather than reducing cell viability, miR-26a inhibition increased viability, in some cases significantly (for example at 24 h in MDA-MB-468). This effect was partially reverted when the mimetic and miR-26a inhibitor were combined (Figure 2). These data support a role for miR-26a in breast cancer cell viability/apoptosis pathways acting as a tumor suppressor.

### 2.2. Effect of miR-26a on Cell Migration

**Figure 3:**
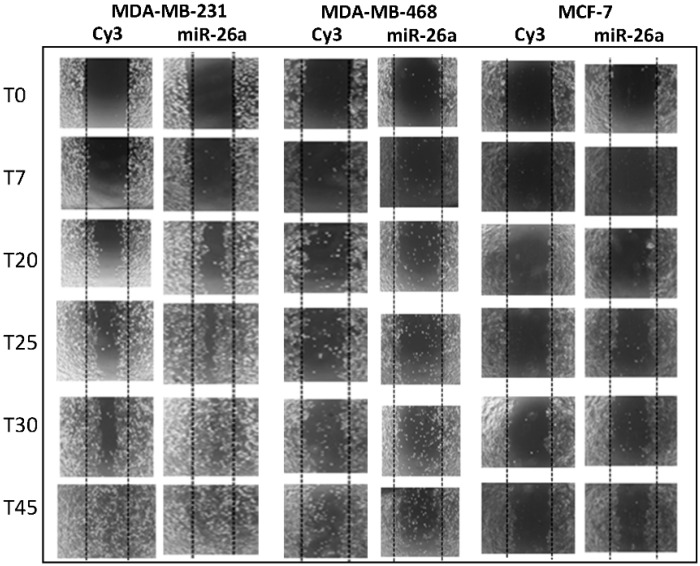
Wound healing cell migration assay comparing cells transfected with 50 nM miR-26a or CY3 (control). Cells transfected with miR-26a closed the wound before the CY3-transfected cells (24 vs. 30 h).

To evaluate the physiological impact of miR-26a regulation we also studied the migration capacity of MDA-MB-231, MDA-MB-468, and MCF-7 cell lines when miR-26a was overexpressed in a wound-healing assay. From 25 h, cell migration was higher in MDA-MB-231 and MDA-MB-468 cells transfected with the miR-26a mimetic compared to control cells, and the effect was more dramatic at 45 h, especially in MDA-MB-231 cells (Figure 3).

### 2.3. Evaluation of Potential miR-26a Targets

We searched for and selected theoretical miR-26a target-genes in the miRTarBase and used DAVID Bioinformatics Resources and miRBase. From 950 potential targets detected in the miRTarBase, we selected 11 based of their relevance to cancer and cell viability and apoptosis processes: *CASP3*, *CCNE2*, *ABL2*, *APAF1*, *XIAP*, *BCL-2*, *PTEN*, *p53*, *E2F3*, *CDC25A*, and *BCL2L1* ligand (Table 1). We differentiate between theoretical and proven targets, and note if the targets have been validated in cancer.

**Figure 4:**
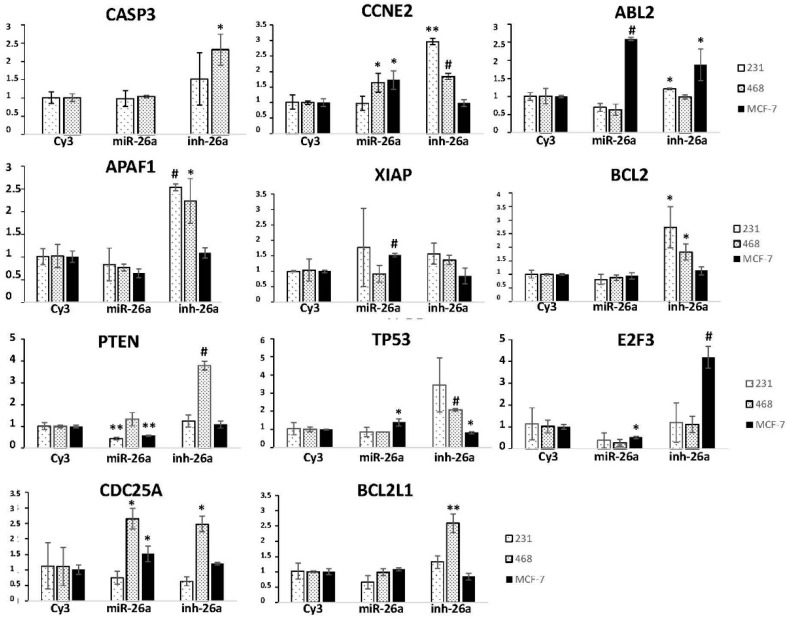
Gene expression of different miR-26a targets four days after transfection with either 50 nM miR-26a or its inhibitor in MDA-MB-231, MDA-MB-468, and MCF-7 cell lines, as measured by RT-qPCR. CY3: CY3 control miRNA. Error bars represent triplicate experiments. (Student *t* test: * *p* < 0.05; ** *p* < 0.005; # *p* < 0.001).

We evaluated the expression of these 11 genes in the three breast cancer cell lines, after transfecting them with a miR-26a mimetic or inhibitor, by RT-qPCR. *CASP3* expression significantly increased (*p* = 0.012) in the MDA-MB-468 cell line, and *CCNE2* expression significantly increased in both the MDA-MB-231 (*p* = 0.002) and MDA-MB-468 (*p* = 0.0008) cell lines after transfection with the miR-26a inhibitor (Figure 4); in MDA-MB-231 cells *ABL-2* was diminished after miR-26a transfection (*p* = 0.06) and increased after its inhibition (*p* = 0.027; Figure 4); *APAF1* expression was higher in MDA-MB-231 (*p* = 0.00015) and MDA-MB-468 (*p* = 0.04) cells with miR-26a inhibition, and decreased in MCF-7 cells upon mimetic transfection (not significant); *XIAP* expression increased with miR-26a inhibition in the MDA-MB-231 and MCF-7 cell lines, significantly in the last (*p* = 0.00025); *BCL-2* increased in the MDA-MB-231 (*p* = 0.018) and MDA-MB-468 (*p* = 0.023) cell lines in the presence of the miR-26a inhibitor; *PTEN* expression diminished upon miR-26a transfection in the MDA-MB-231 (*p* = 0.006) and MCF-7 (*p* = 0.002) cell lines, and significantly increased when miR-26a was inhibited in MDA-MB-468 cells (*p* = 2.45 × 10^−5^); miR-26a inhibition increased *TP53* expression in the MDA-MB-468 cell line (*p* = 0.0009); *E2F3* expression diminished in all three cell lines after miR-26a transfection and significantly increased in MCF-7 cells when miR-26a was inhibited (*p* = 0.0005); both *CDC25A* and *BCL2L1* expression significantly increased after inhibiting miR-26a in the MDA-MB-468 cell line (*p* = 0.024 and *p* = 0.001, respectively) (Figure 4).

For us, *PTEN* and *E2F3* downregulation after miR-26a transfection was the most relevant finding because it suggests that this miRNA directly targets genes; this was especially interesting for *PTEN* as it has proven relevance in cancer processes. Thus, we focused on studying the implications of miR-26a in the modulation of this gene in the context of breast cancer.

### 2.4. Phosphatase and Tensin Homolog (PTEN) Regulation by miR-26a

**Figure 5:**
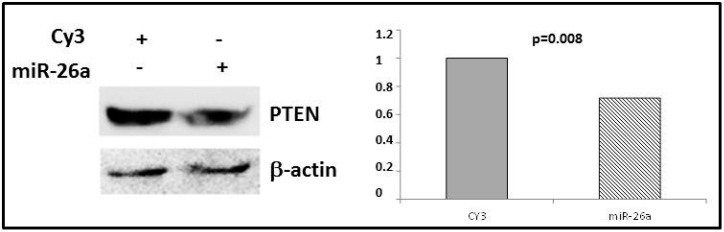
PTEN protein expression was measured by Western blot after transfection with miR-26a or CY3 in MDA-MB-231 cell line. β-Actin was used as a control.

In order to validate *PTEN* as a miR-26a target in breast cancer, we transfected miR-26a mimetic into the MDA-MB-231 cell line and analyzed PTEN protein expression. Western blot analysis showed that levels of this protein significantly decreased (by 35.2% vs. control, *p* = 0.008) after miR-26a overexpression (Figure 5), in concordance with downregulation of this gene after miR-26a transfection (Figure 4).

### 2.5. Effect of Metformin on Breast Cancer Cells

**Figure 6:**
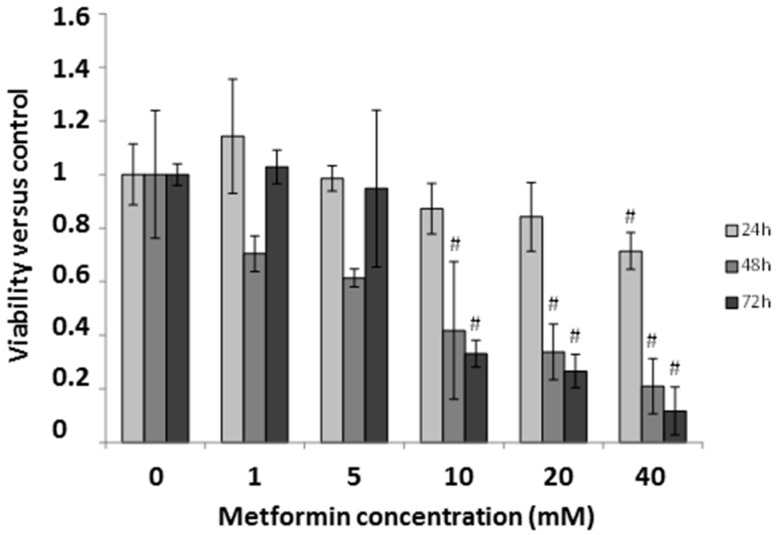
The effect of metformin on MDA-MB-231 cell line viability at different time-points and concentrations. Error bars represent the standard deviation of triplicate experiments. Statistically significant differences are shown for the comparison between treated cells and the control (not treated) at respective time-points (Student *t* test: # *p* < 0.001).

**Figure 7:**
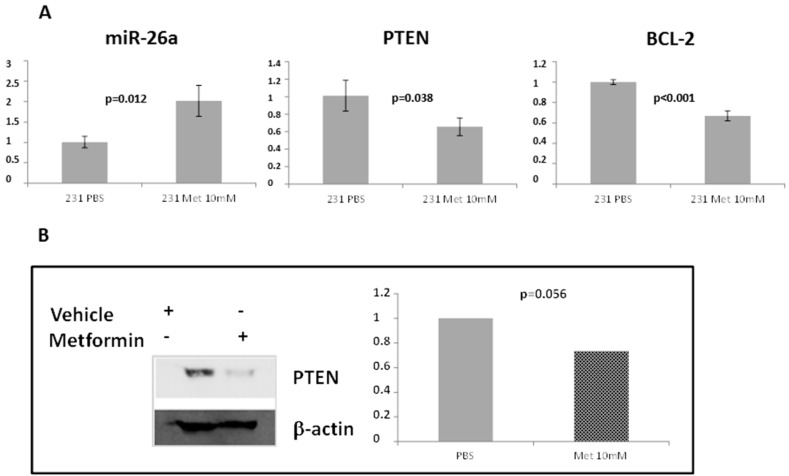
(**A**) Expression of miR-26a and two of its targets in MDA-MB-231 measured by RT-qPCR four days after treatment with 10 mM metformin or vehicle (PBS). Error bars represent triplicate experiments; (**B**) PTEN protein expression measured by Western blot. MDA-MB-231 was treated with 10 mM metformin or PBS, and β-actin was used as a control.

In order to study if metformin induces miR-26a overexpression in breast cancer, as previously described in renal [21] and pancreatic [22] cancer, we tested mRNA and protein expression in metformin-treated MDA-MB-231 cells. First we assessed the effect of metformin on cell viability using a MTT viability test at five different concentrations (1, 5, 10, 20, and 40 mM) at 24, 48, and 72 h of metformin treatment. At concentrations of 10 mM or higher, metformin decreased cell viability at 48 and 72 h (58% and 66% decrease, respectively) (Figure 6). We further analyzed the effect of metformin on the expression of miR-26a and its proposed targets, *PTEN* and *BCL-2*. MiR-26a was significantly increased after treating the cells with metformin (*p* = 0.012), however, both its potential targets showed a significant decrease in expression with the same treatment (*p* = 0.038 and *p* < 0.001 for *PTEN* and *BCL-2*, respectively) compared to non-treated cells (Figure 7A). PTEN protein levels were also lower after treatment with the drug (Figure 7B). These data correlate with our results from RT-qPCR.

**Figure 8:**
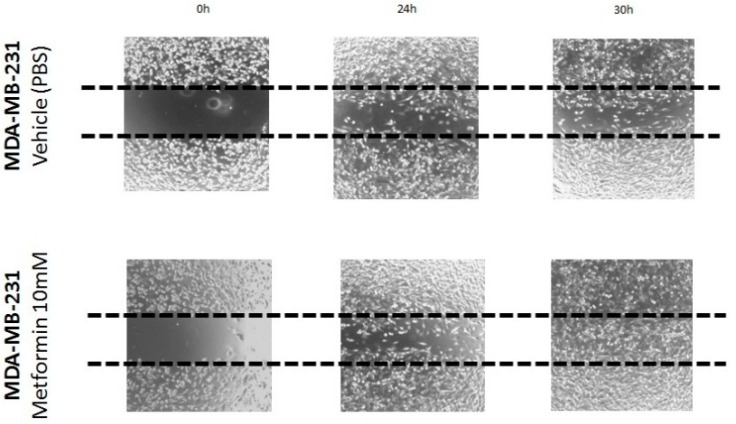
Wound healing cell migration assay comparing metformin-treated (10 mM) MDA-MB-231 cells with non-treated cells at 24 and 30 h. (magnification 100×).

Finally, cell migration during metformin treatment was also checked using a wound healing assay to see if treatment with this drug reproduced the effects seen with miR-26a. Treating MDA-MB-231 cells with metformin increased migration, as shown by faster gap-closing at 24 and 30 h compared to non-treated cells (Figure 8).

### 2.6. Effect of Metformin through miR-26a on Breast Cancer Cells

**Figure 9:**
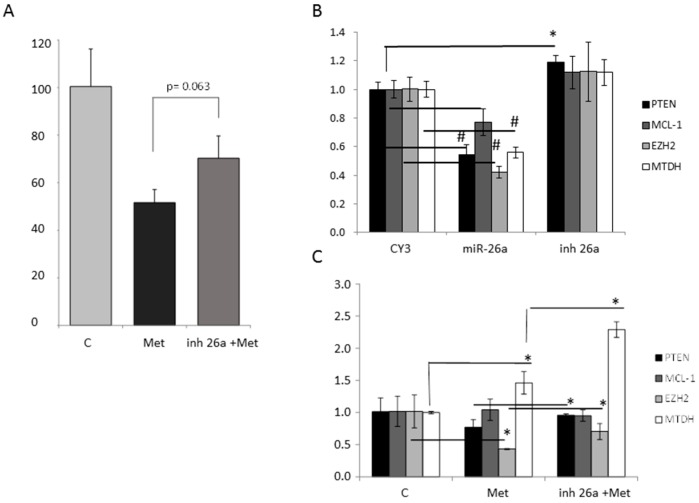
The effect of metformin through miR-26a on MDA-MB-231 cells. (**A**) Cell viability at 48 h after 10 mM metformin treatment with or without the miR-26a inhibitor (50 nM); (**B**) Gene expression of miR-26a targets after transfection with either 50 nM miR-26a mimetic or its inhibitor, as measured by RT-qPCR. CY3: CY3 control miRNA; (**C**) Expression of miR-26a targets measured by RT-qPCR after treatment with 10 mM metformin (in presence or not of miR-26a inhibitor) or vehicle (PBS). Error bars represent triplicate experiments. (Student *t* test: * *p* < 0.05; # *p* < 0.001).

To clarify if the effect of metformin is mediated by upregulation of miR-26a we performed viability and expression analysis combining the miR-26a inhibitor and metformin. The cell viability reduction induced by metformin was partially rescued by miR-26a inhibitor (Figure 9A). We further analyzed the effect of metformin on the miR-26a proposed targets: *MCL-1*, *EZH2*, and *MTDH*. *MCL-1* (myeloid cell leukemia 1) is a pro-survival member of the Bcl-2 (B-cell CLL/lymphoma 2) family [23], *MTDH* facilitates malignant transformation of normal immortal cloned rat embryo fibroblast cells [24], and *EZH2* promotes anchorage-independent growth and invasion of immortalized human mammary epithelial cells [24].

*MCL-1*, *EZH2*, and *MTDH* showed significantly decreased expression (Figure 9B) after transfection with miR-26a mimetic (*p* = 0.02, *p* = 0.0004, and *p* = 0.0004, respectively) as was seen also for PTEN (Figure 4 and Figure 9B). Metformin significantly reduced *EZH2* expression (*p* = 0.02) and the combination of miR-26a inhibitor with the drug significantly reversed the expression levels of *EZH2* and PTEN (*p* = 0.019 and *p* = 0.05 respectively) (Figure 9C).

## 3. Discussion

Evidence for the implication of miRNAs in cancer processes has been growing over the last decade, and many miRNAs have been described as being deregulated in cancer [25]. Consequently, investigation focusing on these small RNAs has increasingly focused on their therapeutic uses. Hence, several strategies have been designed based on miRNA inhibition or enhancement by ectopic expression [26].

We focused on miR-26a because it has been described to play an important role in several cancers, including hepatocellular carcinoma and lung and breast cancer [23,27,28,29]. Furthermore, some authors suggest that metformin, already used to treat diabetes, may modulate the expression of miR-26a. This miRNA is reported to be a tumor suppressor [23,24,27] whose increase leads to better outcomes in tamoxifen-treated breast cancer metastasis patients [30]. However, in lung cancer elevated miR-26a levels have been related to higher levels of tumor cell migration and invasion [28]. Low levels of miR-26a have been associated with TNBC [29] and with stimulating proliferation in ER^+^ breast cancer [31], and miR-26a expression levels have been associated with lymph node metastases in breast [29] and lung cancer [28]. Here we aimed to elucidate the role of miR-26a in modulating TNBC and ER^+^ breast cancer cell viability. Furthermore, we evaluated some of its targets, and finally, we assayed the effect of metformin on miR-26a and these targets.

The use of drugs already on the market for new medical applications significantly streamlines their incorporation into the clinical armamentarium. Therefore, compounds already approved for certain treatments are being re-evaluated to discover their mechanisms of action and to search for possible new therapeutic applications. Metformin, a compound approved for treating type II diabetes, is being evaluated in cancer with the rationale that the incidence of breast cancer is decreased in diabetic patients [7,8] and the risk of metastasis and death by cancer is reduced in breast cancer patients treated with this drug [32,33,34].

The molecular mechanisms of metformin in diabetes control are not completely understood; activation of AMP-activated protein kinase (AMPK), inhibition of the mitochondrial respiratory chain (complex I) and mitochondrial glycerol-3-phosphate dehydrogenase, and a reduction in protein kinase A (PKA) activation have all been proposed as potential mechanisms [9]. The mechanisms by which metformin affects cancers are also unknown, although a large number of publications have shown that metformin could exert its antitumor effect by targeting AMPK/mTOR, anti-inflammatory, cell cycle/apoptosis, insulin/IGF-1R, and angiogenesis pathways in cancers [35,36,37,38]. It has also been shown that it can inactivate cells similar to breast cancer stem cells [39]. Metformin has potent growth-inhibitory and proapoptotic effects in pancreatic cancer [40], and several authors suggest that its biological effects are mediated through miRNA expression [22,40]. Some authors believe that metformin inhibits proliferation by upregulating miR-26a expression which consequently downregulates the targets of this miRNA [21]. Yang et al. [21] first described the involvement of miR-26a and its targets in the metformin mechanism of antitumor action in renal cancer.

Our results confirmed previous data [23] in which miR-26a overexpression reduced cell viability, which was rescued with a miR-26a inhibitor to reverse the effect of the mimetic. Although this effect was slight, it reinforces the potential importance of miR-26a in cell viability/apoptosis processes in breast cancer. We detected a bigger reduction in cell viability in MCF-7 (luminal/ER^+^) cells than in the MDA-MB-468 and MDA-MB-231 (TNBC) cell lines. After evaluating miR-26a expression in different mammary cell line subtypes, one group reported that this miRNA is highly expressed in non-cancerous mammary cell lines but at lower levels in some breast cancer cell lines, in particular TNBC cells [29]. Differences in miR-26a expression in distinct breast cancer subtypes may also lead to different effects when it is exogenously overexpressed or downregulated. Similarly, other authors showed that miR-26a expression is higher in ER^+^ breast cancer [29]. In contrast with its effect on cell viability, miR-26 has been identified as a key mediator of estrogen-stimulated cell proliferation in ER^+^ breast cancer cells [31]. Furthermore, miR-26a seems to be strongly implicated in regulating ER^+^ breast cancer. Chen et al. [27] showed that miR-26a significantly downregulates ERα and prevents the stimulation of hepatoma cell growth by E2. Moreover, in MCF-7 cells, transient transfection of miR-26a initiates apoptosis [24].

Using bioinformatics tools we selected some theoretical miR-26a targets based on their relevance in cancer and their implication in viability/apoptosis processes (in which we and others have found miR-26a to be involved). Interestingly, *PTEN* and *E2F3* were downregulated after transfection with the miR-26a mimetic; PTEN is one of the most commonly mutated tumor suppressors in cancer and has been shown to negatively regulate the AKT/PKB signaling pathway, favoring tumor development and progression. Our data agree with studies showing that PTEN is a miR-26a target in glioma [41,42] and in lung cancer [28]; we also showed that PTEN is downregulated at the protein level in breast cancer cells overexpressing miR-26a.

The evaluation of metformin cellular effects reveals, according to other authors in kidney, pancreas and renal cancer [21,22], that the drug reduces cell viability in a dose-dependent manner in the breast cancer cell line MDA-MB-231 and that its administration increases miR-26a and reduces *BCL-2* and *PTEN* expression. However, although the beneficial effects of metformin on breast cancer patient survival rates has been described by several authors [32,33,34,43,44,45,46], little is known about the mechanism. We show here that metformin up-regulated miR-26a and also downregulated its direct target *PTEN*. It is difficult to explain how metformin can have an anti-proliferative effect since *PTEN* is a tumor suppressor gene. Trying to understand how the *PTEN* inhibition by miR-26a can result in an anti-proliferative effect, we checked the effect of miR-26a overexpression in some additional targets of this miRNA. We identified *EZH2*, a miR-26a target that is downregulated by the miRNA and by metformin. *EZH2* is a bona fide oncogene and acts as a dual function transcription regulator (not only repressor but also activator) [47] by converging on the methyltransferase-activity silencing tumor suppressor genes, which are implicated in neoplastic development and the transactivation property-activating genes involved in the late-stage process of cancer [48,49]. This gene has been implicated in promoting anchorage-independent growth and invasion of immortalized human mammary epithelial cells [24]. Retrospective studies from clinical breast cancer patients indicate that high expression of *EZH2* is associated with short survival [50]. Therefore, it can be one of the effectors involved in the decrease in cell viability after treatment with metformin and overexpression of miR-26a and thus justify their anti-proliferative effect.

It has been shown that metformin reduces cell migration. In general, we confirmed these data, although we observed slightly increased migration in the TNBC-model MDA-MB-231 cell line when the cells were treated with either a miR-26a mimetic or metformin. Our data are consistent with observations about the different behavior of breast cancer subtypes when miR-26a was upregulated. Boning Liu et al. [28] demonstrated that miR-26a increases lung cancer cell migration and the risk of metastasis by modulating activation of the AKT pathway by suppressing *PTEN*, data which agrees with our results in MDA-MB-231 cells. *PTEN* loss promotes cell migration in cancer cells, as previously described in breast cancer [51,52,53]. Simultaneous downregulation of *PTEN* and *DLC1* in MCF-7 cells does not enhance cell proliferation, however, enhances cell migration [51]. *DLC1* is negatively regulated by miRNAs in colorectal cancer [54,55], however, the possible *DLC1* regulation by miR-26a has not been evaluated.

We believe that *PTEN* inhibition by metformin via miR-26a could explain the increased cell migration under the treatment, but the metformin antitumor activity must be due to other miR-26a targets. We checked the gene expression levels of *MCL-1*, *MTDH*, and *EZH2* which are proven targets of miR-26a in breast cancer and could be responsible of its anti-proliferative effect. We observed that *EZH2* expression under metformin treatment was also lower than in non-treated cells, suggesting that metformin antitumor effect could involve this gene [23,24]. We also checked *EZH2* expression under metformin treatment when transfecting miR-26a inhibitor and their levels were partially rescued, suggesting not only that metformin antitumor effect can be through this gene, but also that it happens via miR-26a overexpression. 

## 4. Materials and Methods

### 4.1. Cell Lines and Culture

The human MDA-MB-231 and MDA-MB-468 (both TNBC), and MCF-7 (luminal) cell lines were obtained from ATCC (ATCC, Manassas, VA, USA). The TNBC cell lines were maintained in DMEM/F12 medium with 10% fetal bovine serum (FBS) and MCF-7 cells were maintained in DMEM with 10% FBS; all the cell lines were cultured with 1% antibiotics (100 U/mL penicillin and 100 mg/L streptomycin) and were maintained in a humidified atmosphere with 5% CO_2_ at 37 °C.

### 4.2. Cell Viability Assay

Cell viability was measured using a MTT-based cell growth [56] determination kit (#GDC1; Sigma-Aldrich, St. Louis, MO, USA). At the indicated intervals, MTT was added to each well and incubated for four hours at 37 °C. The medium was then carefully discarded and 50 µL MTT solvent was added to each well to dissolve the formazan crystals. Purple formazan crystals are formed from yellow MTT by succinate dehydrogenase in viable cells. The absorbance at 570 and 690 nm was measured using a microplate spectrophotometer and the percentage of surviving cells from each group relative to controls were calculated in triplicate.

For the viability assays, cells were seeded at an initial density of 3 × 10^3^ cells/mL in a 96-well plate and incubated with medium, transfection reagents Cy3 miRNA and 50 nM miR-26a mimic or inhibitor for different time periods at 37 °C. For metformin (Sigma-Aldrich) treatment, 24 h after seeding, the cells were treated with metformin (0, 1, 5, 10, 20, 40 mM) and viability was measured at 24, 48, and 72 h as described above.

### 4.3. MicroRNA Transfection

To increase or reduce the miR-26a levels, 50 nM of miR-26a mimetic miRNA or inhibitor (Applied Biosystems, Foster City, CA, USA) were transfected using a TransIT-X2TM [57] polymeric non-liposomal system (Mirus Bio Corporation (Madison, WI, USA) following the manufacturer’s instructions; 50 nM of CyTM3 dye-labeled Pre-miRTM negative control was transfected as a negative control.

### 4.4. Analysis of miRNA and mRNA Expression by Quantitative Real-Time PCR

RNA from cell lines was harvested using a miRNA isolation kit (mirVana, Ambion, Inc., 2130 Woodward Street, Austin, TX, USA) for miRNA and a TRIzol procedure for mRNA. The concentration and quality of the extracted RNA were determined by measuring OD_260_ and the OD_260_:OD_280_ ratio. First, 150 ng RNA were reverse transcribed to cDNA with specific stem-loop RT primers using a TaqMan microRNA reverse transcription kit (Applied Biosystems) for miRNA, and then 150 ng RNA were reverse transcribed to cDNA with random primers using a high-capacity cDNA reverse transcription kit (Applied Biosystems) from TaqMan for mRNA samples. RT-qPCR was performed using an ABI 7900HT fast RT-qPCR system and a TaqMan universal master mix (Applied Biosystems). All the primers were obtained from the TaqMan miRNA and mRNA assay kits (Applied Biosystems). The endogenous microRNA RNU43 [58] was used as an internal control for miRNA expression, and the housekeeping gene *GAPDH* was used as an internal control for mRNA expression.

### 4.5. Western Blot Assays

Cells were lysed in a radio-immunoprecipitation assay (RIPA) lysis buffer containing protease inhibitor. Protein concentrations were determined using the Lowry-Folin method. Following SDS-PAGE separation, 50 µg of protein were transferred to polyvinylidene difluoride membranes (Bio-Rad). The membranes were blocked in tris-buffered saline (TBS) containing 5% non-fat milk and were subsequently incubated at 4 °C overnight with specific PTEN (1:500 in TBS with 0.1% Tween 20 (TBS-T) and 5% bovine serum albumin (BSA)) or β-actin (1:1000 5% non-fat milk in TBS-T) primary antibodies, and then washed repeatedly for 5 min with 1% BSA-TBS-T followed by incubation with anti-rabbit-horseradish peroxidase (HRP; 1:2500 5% non-fat milk in TBS-T) or anti-mouse-HRP (1:7500 5% non-fat milk in TBS-T) secondary antibodies for 1 h at room temperature (all antibodies were from Cell Signaling, Beverly, MA, USA). ECL reagent (Amersham Life Science, Piscataway, NJ, USA) was used for detection and the membranes were developed in an ImageQuant LAS 4000 (GE Healthcare Life Sciences, Princeton, NJ, USA).

### 4.6. Wound Healing Assay

Cell migration was examined using a wound-healing assay [59]. Cells were cultured in six-well plates to 100% confluence. A plastic pipette tip was used to generate a wound area across the center of each well and after the wells were washed with PBS the medium was replaced and they were allowed to migrate. Micrograph images were taken with a microscope at 40× magnification at the indicated time points. All these experiments were repeated in triplicate.

### 4.7. Bioinformatic Databases

miRBase database (http://www.mirbase.org/) was used to obtain information about miR-26a, and miRTarBase (http://mirtarbase.mbc.nctu.edu.tw/) was used to look for its gene targets. The first 950 genes were selected and analyzed in DAVID [60] (the database for annotation, visualization, and integrated discovery) bioinformatics resources to select targets involved in the most relevant pathways for cancer such as proliferation and apoptosis.

### 4.8. Statistical Analysis

The data were presented as the mean ± standard deviation (SD) of the triplicate experiments. We analyzed the significance of any difference between the control and treatment groups using the Student *t* test and the level of statistical significance was set at 95% confidence (*p* < 0.05).

## 5. Conclusions

We have confirmed the anti-proliferative effect of metformin in breast cancer. Our results suggest that upregulation of miR-26a and downregulation of some of these miRNA-targets are part of the action mechanisms of this drug. miR-26a is at least partially responsible for the metformin antineoplastic effect in breast cancer, even in triple negative breast cancer where there is no treatment other than chemotherapy, and this drug could result in a real improvement in treating the disease.

## Figures and Tables

**Table 1:** Theoretical and demonstrated miR-26a selected gene targets obtained with miRTarBase, and selected by DAVID Bioinformatics Resources and miRBase. Demonstrated targets are experimentally proveN targets by techniques such as RT-qPCR or Western Blot, although none of them are proven in breast cancer.

miR-26a Target Genes	
*CASP3*	Theoretical
*CCNE2*	Demonstrated
*ABL2*	Theoretical
*APAF1*	Theoretical
*XIAP*	Theoretical
*BCL2*	Theoretical
*PTEN*	Demonstrated
*TP53*	Theoretical
*E2F3*	Theoretical
*CDC25A*	Theoretical
*BCL2L1*	Theoretical
